# Laterally Actuated Si-to-Si DC MEMS Switch for Power Switching Applications

**DOI:** 10.3390/mi15111295

**Published:** 2024-10-24

**Authors:** Abdurrashid Hassan Shuaibu, Almur A. S. Rabih, Yves Blaquière, Frederic Nabki

**Affiliations:** Department of Electrical Engineering, École de Technologie Supérieure, Université du Québec, Montréal, QC H3C 1K3, Canada; almur-abdelkreem-saeed.rabih.1@ens.etsmtl.ca (A.A.S.R.); yves.blaquiere@etsmtl.ca (Y.B.); frederic.nabki@etsmtl.ca (F.N.)

**Keywords:** electrothermal actuator, microelectromechanical systems (MEMS), PiezoMUMPs, DC MEMS switch, Si-to-Si contact resistance

## Abstract

Electrothermal actuators are highly advantageous for microelectromechanical systems (MEMS) due to their capability to generate significant force and large displacements. Despite these benefits, their application in reconfigurable conduction line switches is limited, particularly when employing commercial processes. In DC MEMS switches, electrothermal actuators require electrical insulation between the biasing voltage and the transmission line to prevent interference and maintain the integrity of the switch. This work presents a chevron-type electrothermal actuator utilizing a stack of $SiO_{2}/$ Al thin films on a silicon (Si) structural layer beam to create a DC MEMS switch. The design leverages a thin film Al heater to drive the actuator while the $SiO_{2}$ layer provides electrical insulation, suppressing crosstalk with the Si layer. The electrical contact resistance of a Si-to-Si interface was evaluated by applying a controlled current and measuring the resultant voltage. A low contact resistance of 150 Ω was achieved when an initial contact gap of 2.52 μm was closed using an actuator with an actuation voltage of 1.2 V and a current of 205 mA, with a switching speed of less than 5 ms. Factors such as the contact force, the temperature, and the residual device layer etching angle significantly impact the Si-to-Si contact resistance and the switch’s longevity. The switch withstands a breakdown voltage up to 350 V at its terminal contacts. Thus, it will be robust to self-actuation caused by unwanted voltage contributions, making it suitable for high-voltage and harsh environment applications.

## 1. Introduction

In recent years, reconfigurable, integrated, and miniaturized switches have been in high demand in order to reduce the complexity of power switching and fault protection systems in the transport industry [1,2,3]. Microelectromechanical systems (MEMS) switches/relays combine the benefits of solid-state switches, such as their compact size, low energy consumption, low cost, and compatibility with other integrated circuits on the same substrate [4]. Most importantly, they mirror the desired characteristics of conventional electromechanical relays through direct physical contact and low leakage currents [5]. Accordingly, MEMS switches can feature low insertion losses, high linearity, high isolation, and wide operation electrical bandwidth [6,7]. This can be advantageous for implementing configurable power networks and other payload systems [8,9,10]. These make switches based on MEMS technology an interesting and active area of research. However, despite the progress of DC MEMS switches, the endurance to withstand large currents and breakdown voltages has been a bottleneck [11,12], causing some unsatisfactory reliability issues. These issues include contact stiction, wear and micro-welding, material transfer between the contact, high contact resistance, and dielectric breakdown of the air gap at the off-state [13]. Many of these issues are found in electrostatically actuated switches [13,14]. In this regard, MEMS switches based on thermal actuation stand to be of great interest to many applications such as RF MEMS switches [15,16], optical switches [17,18], and tensile loading [19], due to promising features such as high contact force, robustness, wide travel range, in-plane design flexibility, and low fabrication complexity [20,21,22]. As the actuation mechanism is built on a resistive heating element, it requires a DC current to maintain its active state. This can also be customized to achieve a bistable switch to eliminate the current and power consumption during operation [23]. The thermal actuator’s robustness in environments where high-voltage spikes can be observed is another advantage making this actuation mechanism suitable for applications in the transportation sector, which require high-voltage resilience [24].

Several works on electrothermal MEMS switches for power switching applications using commercial foundry processes provided by MEMSCAP (Crolles, France) were published, mostly using the Metal Multi-User MEMS Process (MetalMUMPS) [8,23,25]. However, that process lacks a silicon (Si) structural layer, which brings challenges with regard to the robustness of the designs for harsh environments. In [26], a study was reported on electrostatically actuated Si-to-Si contact microswitches that were custom-fabricated using an ultraclean encapsulation process and operated within a temperature range of −60 °C–300 °C, but the contact resistance was in the hundreds of kiloohms. In [27], the MEMSCAP Silicon-on-Insulator Multi Users Micromachining process (SOIMUMPS) was utilized to design a structure with a hybrid actuation mechanism. The design used an electrostatic latching voltage of 21 V to keep the switch in an active state. However, it was not possible to isolate the RF signal line from the actuator signal. In [28], a test platform that utilized a V-shaped thermal actuator driven by a thin-film aluminum (Al) heater stacked onto a ${SiO}_{2}$ layer was reported. The actuator was robust against out-of-plane deformation due to its high aspect ratio, with a Si structural layer thickness of 50 µm. However, it required substantial energy consumption equivalent to 200 mA at 3 V to close the contact. Recently, in [29], an electrostatically actuated MEMS switch for microwave band application using the MEMSCAP Piezoelectric Multi-User MEMS Process (PiezoMUMPs) was reported. The authors observed the non-uniformity of the contact with a scallop diameter of 0.33 μm on the sidewall interface. As such, non-ohmic contact was observed and demonstrated. This observation implies that the contact interface can be improved by mechanical polishing.

Previously, in [30], we demonstrated an electro-thermal chevron-type (i.e., V-shape) MEMS actuator fabricated using the PiezoMUMPs process and aimed at power switching applications that require high breakdown voltage, taking advantage of the high-robustness silicon structural layer provided by the process [31]. However, the Si-to-Si contact interface has shown inconsistency in ohmic contact resistance measurement. A close observation of the interface demonstrated scallops that could cause out-of-plane misalignments of the conducting lines upon contact. Other issues observed included the influence of temperature and the possible appearance of a native oxide across the sidewall interface, besides the restricted lifespan of the switch due to the high current density sustained by the heater.

Accordingly, this work further investigates the design, fabrication, and characterization of a V-shape MEMS actuator with an improved force, using the PiezoMUMPs process. The actuator is integrated with a shuttle to implement a direct contact (DC) switch aimed at power switching applications and high-voltage resilience. Additionally, this work offers potential engineering approaches to enhance the understanding of the impact of silicon sidewall morphology and its negative effects on the formation of reliable ohmic contacts. The study also investigates the effect of the contact temperature on the contact resistance by implementing a near zero-temperature actuation test, i.e., by manual contact operation through a micro-needle.

This paper is structured as follows: Section 2 presents the proposed design along with the simulation results. Section 3 provides a brief overview of the prototype fabrication process. Section 4 details the experimental results of the fabricated design, including the DC characterization of the actuator, the *I–V* curve of the Si-to-Si contact interface, the etching angle profile, and the switching speed of the actuator. Section 5 discusses the influence of the sidewall contour on the contact interface of the MEMS switch. Finally, Section 6 concludes the paper.

## 2. Design and Simulation

### 2.1. Design Configuration

**Figure 1:**
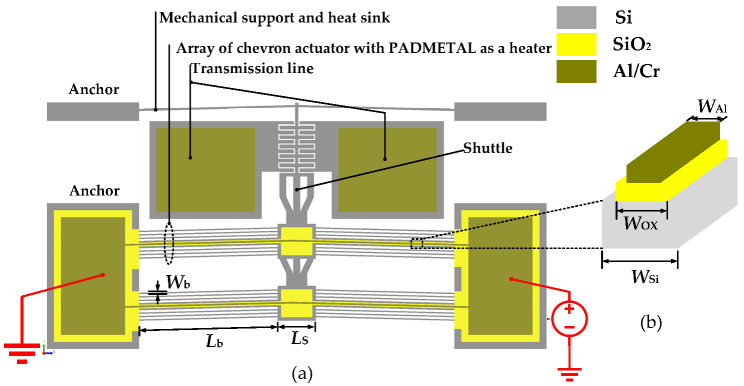
Schematic illustration of the thermally actuated DC MEMS switch: (**a**) top view and (**b**) cross-section of the Al heater on top of the Si layer.

The schematic shown in Figure 1 illustrates the proposed concept of an in-plane electrothermally actuated Si-to-Si DC MEMS switch. In this work, variations in the widths of different layers are determined by fabrication constraints, which require specific enclosure distances between the layers to ensure proper functionality and manufacturability. The main part of the design is a chevron type electrothermal actuator driven by a heater built from the Padmetal layer made of aluminum (Al) and chromium (Cr), stacked onto a silicon Si structural layer via a thin silicon dioxide SiO_2_ layer. The SiO_2_ layer, positioned between the Al and Si layers, provides electrical isolation between the switch transmission line and the driving heater current.

Typically, the chevron actuator features a symmetric geometry that is composed of an array of beams slanted at a tilted angle, *β*, in such a way that the generated heat enables lateral motion due to joule heating and thermal expansion.

To reduce the out-of-plane deformation, only one pair of the chevron beams is covered with the SiO_2_/Al-Cr layer to act as a heater, allowing the remaining chevron beams to have a reduced width of 6 μm, as depicted in Figure 1. This is advantageous, as it reduces the stiffness and serves as a heat sink to reduce the contact temperature. In addition, these narrower beams reduce the high impact of the lower aspect ratio of the heater beam (i.e., the Si thickness, $t_{Si}$ = 10 μm, is significantly smaller than the Si width, $W_{Si}$ = 21 μm) due to fabrication process constraints. Figure 1b illustrates the patterning of the silicon chevron beams with SiO_2_/Al-Cr layers, having a total width of 21 μm. In this configuration, the SiO_2_ layer encloses the side edges of the Al-Cr layer by 3 μm. The design includes an air gap of 3 μm between the switch contacts, which the actuator must traverse to bring the shuttle comb structure into contact with the transmission line structures, thereby closing the switch. In addition, the multilayered structure is vulnerable to intrinsic stress gradients due to the wide coefficient of linear thermal expansion (CTE) mismatches between the silicon ($\alpha_{Si}$ = 2.6 × 10^−6^/K) and $Al(\alpha_{Si}$ = 23.1 × 10^−6^/K) layers. As a result, the beam’s moment of inertia is smaller for out-of-plane motion compared to in-plane motion, leading to an undesired tendency for the actuator to favor out-of-plane motion during operation [12]. This needs to be carefully assessed in the simulation.

### 2.2. Simulation

The CoventorWare11.1 FEM software package with an electro-thermomechanical solver was utilized to study the displacement, temperature, and current of the heater over an applied voltage range. In the solid mechanics physics, the two anchors of the V-shaped beam of the actuator are set to be fixed boundaries, where the voltage is applied across the anchor points. The room temperature is set at the bottom of the anchors and the rest of the structure is configured to exhibit conductive heat transfer [32]. The designed dimensional parameters in Figure 1 are listed in Table 1.

**Figure 2:**
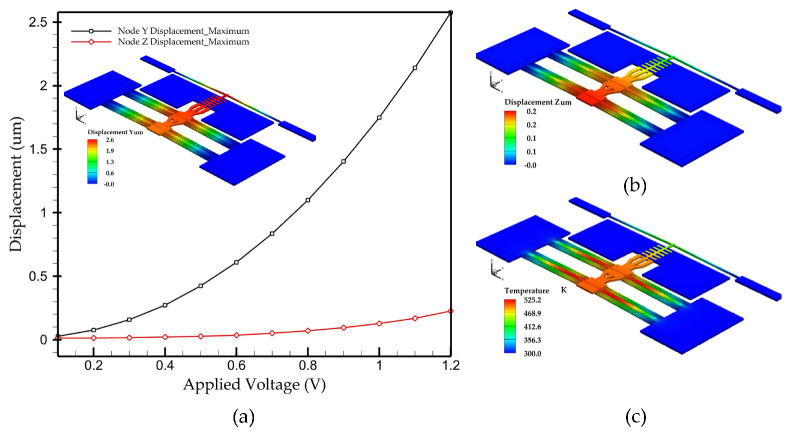
(**a**) Simulation results of the displacement vs. actuation voltage, (**b**) out-of-plane displacement profile, and (**c**) temperature profile of the thermal actuator.

The displacement of the actuator at the shuttle tip for a voltage ranging from 0 to 1.2 V applied across the actuator terminals (i.e., heater) is shown in Figure 2a. This 1.2 V is the typical voltage required to provide in-plane displacement of 2.52 μm to close the initial contact gap. At this displacement, the out-of-plane displacement (misalignment with respect to the fixed parts) was recorded to be 100 nm, as illustrated in Figure 2b. It is deemed acceptable. The maximum generated temperature on the actuator was 525.2 K, as shown in Figure 2c, whereas the recorded temperature on the contact of the switch was 450 K. Note that the actuator can operate only at temperatures that are below the melting point of the heater’s aluminum material, which is 960 K [33].

## 3. Fabrication and Characterization

**Figure 3:**
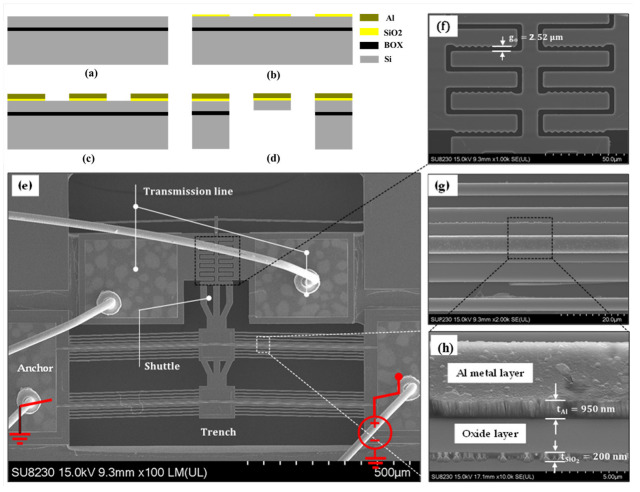
Structure of the fabricated thermally actuated Si-to-Si DC MEMS switch: (**a**–**d**) simplified fabrication process flow, (**e**) SEM of the switch, (**f**) zoomed-in image of the switching contact area, and (**g**,**h**) zoomed-in image of the chevron beam detailing the Al heater and silicon oxide insulating layer.

The switch devices were fabricated following the PiezoMUMPs process technology provided by Science Corporation [34]. The process with a total of five masks begins with a 400 μm-thick handle substrate, an n-type (100) silicon orientation wafer with a 1 ± 0.05 μm buried oxide (BOX) insulation layer, and a 10 μm-thick phosphorus-doped silicon device layer. Figure 3a,d summarize the sequence of steps involved in the fabrication process of the thermally actuated MEMS switch devices. A thermally grown 200 nm-thick $SiO_{2}$ layer is patterned to provide electrical isolation, as shown in Figure 3b. The microheater, composed of a 1 μm-thick Al layer and a 20 nm-thick Cr adhesion layer, is deposited on top of the device structural layer using e-beam evaporation and patterned through a lift-off technique, as shown in Figure 3c. The suspended silicon structural layer in Figure 3d is patterned through front side reactive ion etching (RIE) to remove the thermal silicon dioxide layer and deep reactive ion etching (DRIE) to remove the silicon structural layer. This is followed by coating the front side with a protective polyimide layer and the backside RIE on the silicon dioxide layer. The silicon handle wafer is then removed through DRIE. After the etch is completed, the photoresist is removed, and a wet oxide etch process is then used to remove the SiO_2_ layer in regions defined by the trench mask. The frontside polyimide layer is finally removed using an oxygen dry etch process. The released device cross-section is shown in Figure 3d.

Prior to experimental testing, the fabricated devices were visually inspected using a scanning electron microscope (SEM) model SU6 300, as shown in Figure 3e–h. The measured thickness of the Al layer is ~950 nm compared to the designed value of 1 µm, whereas the designed contact gap of 3 μm was measured to be 2.52 μm. These small discrepancies could be attributed to variations in the deposition process parameters. Figure 3h shows a higher grain boundaries defect for the Al layer compared to the oxide layer.

## 4. Measurements and Results

This section discusses the switch electrical characterization and behavior of the ohmic contact. Initially, the on/off characteristics are measured, followed by an evaluation of the contact resistance during quasi-static actuation. Additionally, the transient response of the chevron actuator upon establishing the ohmic contact is analyzed.

### 4.1. Displacement Measurement

**Figure 4:**
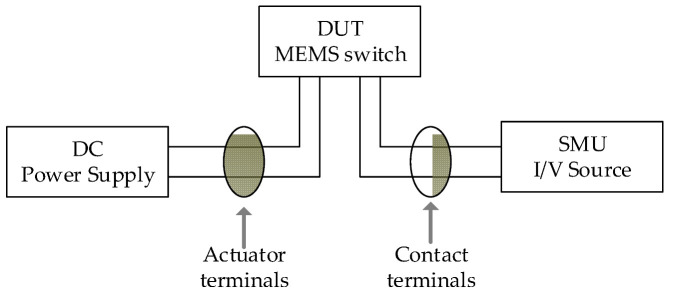
Schematic of the DC measurement setup for MEMS switch characterization.

Figure 4 illustrates the schematic diagram for the DC characterization setup used to measure displacement and contact resistance. The setup includes a Tektronix PWS4205 programmable DC power supply (0–20 V, 5 A), a source meter measurement unit, and a Keyence VHX7000 optical microscope. The device under study is mounted on a ceramic DIP package. The device is wire-bonded using gold wires. Subsequently, the package is connected into a prototype PCB board for measurement.

**Figure 5:**
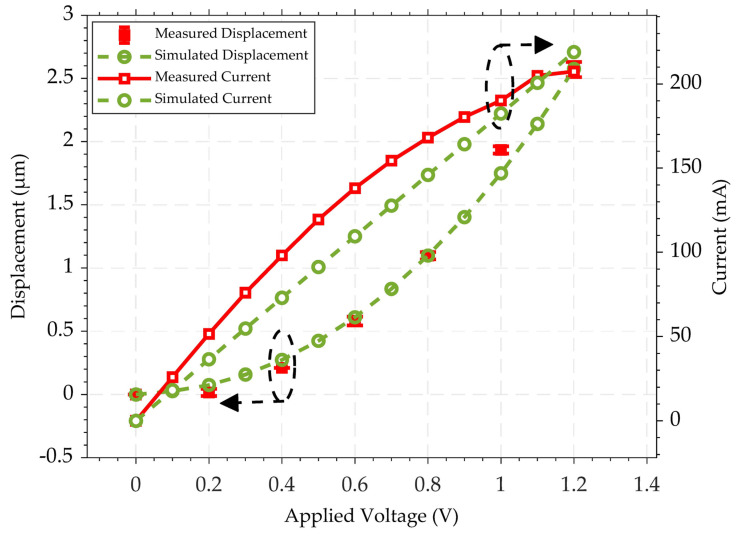
Switch displacement and actuator current as a function of applied heater voltage.

To verify the functionality of the fabricated devices, the static response of the chevron thermal actuator driven by the Al heater was examined. The displacement of the thermal actuator’s contact tip was measured by capturing its motion at each increment of the biasing voltage using an optical microscope until the gap was entirely closed at an average measured displacement of 2.52 μm. The actuation voltage and current (*I–V*) necessary to close the switch gap were found to be 205 mA and 1.2 V, respectively, as shown in Figure 5. The observed difference between the measured and simulated *I–V* curves in Figure 5 is mainly due to the simulation model assuming ideal electrical conditions. However, real-world factors, including environmental conditions and fabrication tolerances, such as variations in the thickness and width of the aluminum layer across the interconnect geometry, introduce deviations that are not captured by the simulation.

### 4.2. Contact Resistance Measurement

**Figure 6:**
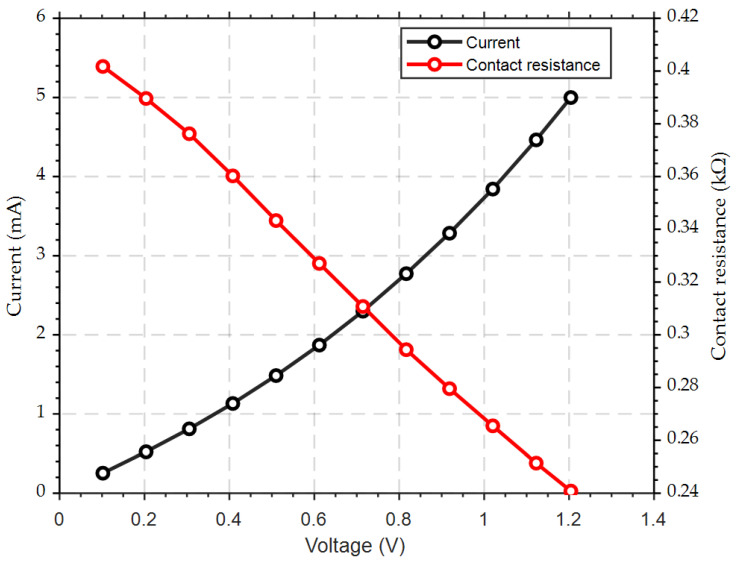
*I–V* plot with the extracted electrical contact resistance of the Si-to-Si contact.

The contact resistance of the MEMS switch was measured using a source meter unit (SMU) after the switching interfaces were closed and mechanically settled. The test setup utilized to determine the contact resistance is depicted in Figure 4. Upon closing the MEMS switch, the initial contact resistance was observed to be around 400 Ω. The current–voltage (*I–V*) characteristics, shown in Figure 6, were obtained with a test limiting a current of 5 mA passing through the switch. Within the tested limited current, the voltage drop across the contact was found to vary due to the variation in the contact resistance, where the initial measured 400 Ω resistance corresponds to a ~0.25 mA testing current and a 0.1 V drop across the contact. At the maximum testing current of 5 mA, the measured contact resistance dropped to only 240 Ω; therefore, the maximum measured voltage drop was 1.2 V, as shown in Figure 6. This progressive decrease in the contact resistance with the increase in the testing current results in erratic behavior and inconsistencies in the ohmic contact. The initial establishment of the contact resistance suggests that instability may occur when the contact force is below the threshold value, coupled with the high temperature across the shuttle resulting from resistive heating. Notably, this variation in resistance with voltage may be due to the resulting self-heating of the switch causing additional force to be generated by the actuator. This highlights the importance of optimizing both the mechanical force and thermal management to ensure uniform switch operation.

### 4.3. Switching Time

**Figure 7:**
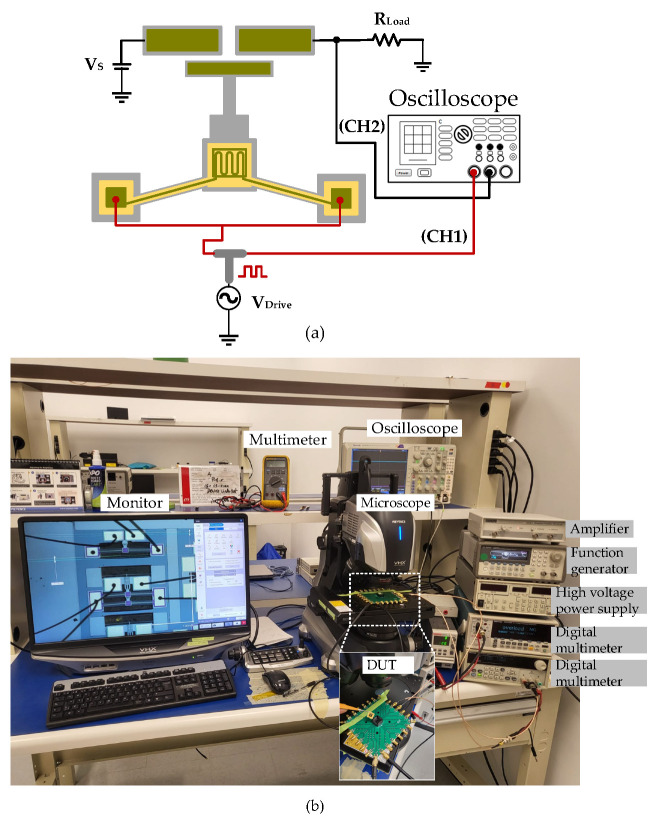
Switching speed test measurement setup: (**a**) schematic and (**b**) photograph.

**Figure 8:**
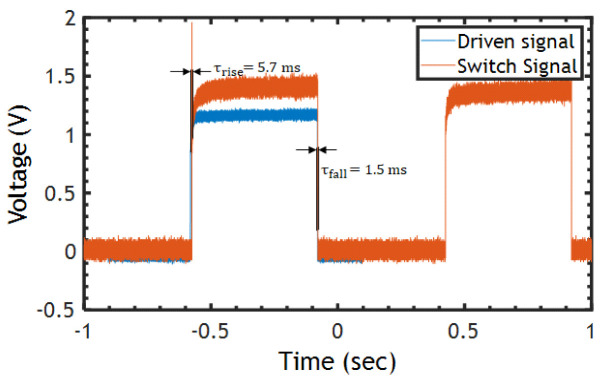
Measured switching speed of the actuator at a maximum range of 2.52 μm.

The dynamic transient response was measured using a square wave signal toggling between 0 and 1 V at a rate of 1 Hz and applied to the Al heater. The switching speed of the proposed MEMS switch is explored in ambient air with a simple test circuit depicted in Figure 7a. $V_{\mathrm{Drive}}$ generates a driving signal for the heater and activates the switch. The switching contact is used as a sensor to detect the response time of the actuator when excited to the desired displacement. Thus, this creates a current path from the supply voltage, $V_{S}$, through the floating contact to the off-the-shelf resistor, $R_{\mathrm{Load}}$. Both $V_{Drive}$ and the voltage across the resistor $R_{\mathrm{Load}}$ are monitored using an oscilloscope. The actual setup used to measure the response time of the switch is shown in Figure 7b. It was observed that the reaction time for a rising edge $\tau_{rise}$ was ~5.7 ms, whereas the falling edge, $\tau_{fall},$ was found to be ~1.5 ms, as measured in Figure 8. The rise time is longer than the fall time, and this is attributed to the Joule heating time constant and the cooling time constant being different. Note that the variability of the voltage across $R_{Load}$ is due to the variation in the contact resistance, as mentioned above. Moreover, potential reasons for the deviation include fabrication imperfections, mainly over-etching and the etching angle during the DRIE process. The high aspect ratio also increases the stiffness and out-plane displacement.

### 4.4. Breakdown Voltage Test

**Figure 9:**
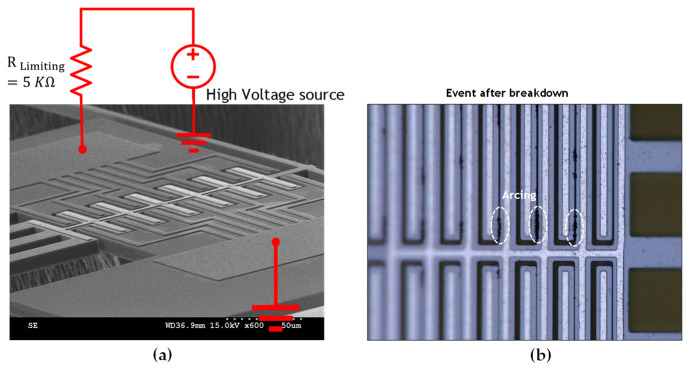
Breakdown voltage measurement: (**a**) test schematic, (**b**) microscope image after breakdown event.

Using a high-voltage source manufactured by Stanford Research Systems (model PS310), the switch was kept in an open state and a high voltage was applied to one of the transmission terminals while the other was grounded, as given in the setup depicted in Figure 9a. The voltage required to break down the structure was visually monitored on the microscope and found to be 350 V, and it is limited by the 2.52 μm air gap between the shuttle and the switch transmission line, which results in arcing, as shown in Figure 9b. The stiffness of the chevron actuator also plays a significant role by preventing self-actuation due to electrostatic forces. This can be enhanced by increasing the gap size at the cost of the larger actuation displacement required to close the switch and, consequently, more actuation power.

## 5. Discussion

**Figure 10:**
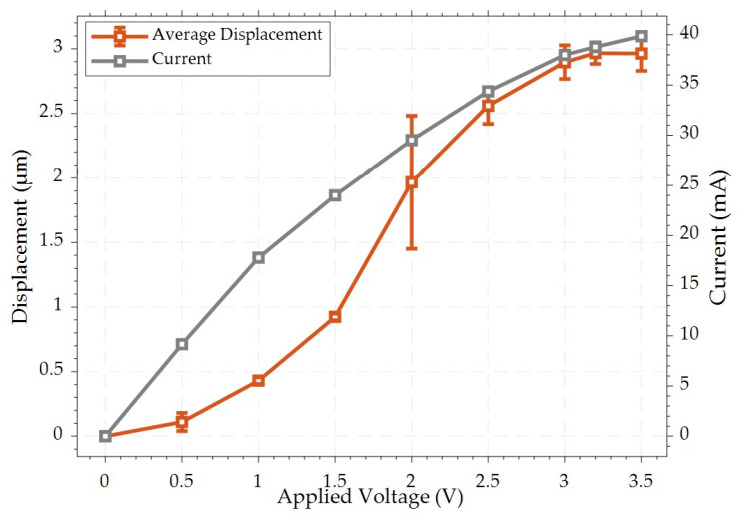
Displacement and current as a function of applied voltage for the previous actuator reported in [30].

**Figure 11:**
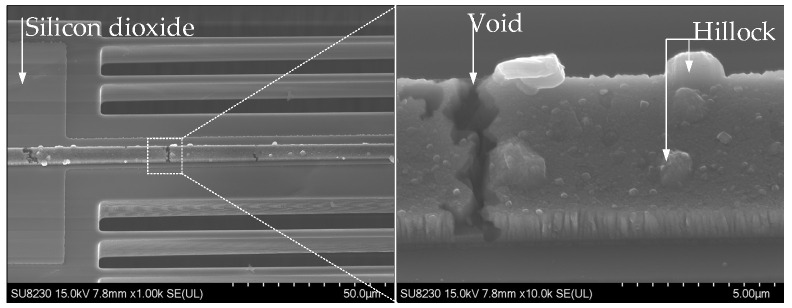
Degradation of aluminum heater over time.

The results presented in the previous section demonstrated that driving the chevron thermal actuator through Joule heating, with a stacked Al layer as the resistive heating element for DC MEMS switching applications, requires an insulating layer between the resistive heating and the transmission line. However, this approach can potentially cause unwanted out-of-plane displacement due to the stress of both insulating and metal layers, which is a trade-off for achieving the desired in-plane motion crucial to this research work. In this study, the effect of the out-of-plane deformation was mitigated by the parallel connection of several narrow Si beams with the stacked (${SiO}_{2}$/Al-Cr) chevron beams. The simulated out-of-plane displacement was found to be 100 nm. Additionally, reducing the width of the narrow beams to 6 µm significantly mitigates the temperature gradient across the actuator’s shuttle, as compared to the local temperature of the actuator beams. The lateral stiffness of the actuator is also mitigated due to the presence of the narrow beams. Furthermore, the stress across the switching contact is mitigated, as no Al layer is deposited on the Si-to-Si contact. Notably, the initial contact resistance, as depicted in Figure 5, showed an improvement over that in our previously reported study [30], where all the pairs of the chevron beams were patterned with a ${SiO}_{2}$/Al-Cr stack. The thermal actuator closes a measured switch gap of 2.5 μm, with resistive heating at 33 mA/2.4 V, as depicted in Figure 10. Despite this, infinite electrical contact resistance (ECR) was observed at this initial setting. Consequently, increasing the heater bias voltage by an additional 0.6 V resulted in achieving a contact resistance of approximately 100 MΩ. Furthermore, as the resistive heating current of the thermal actuator was increased to 42 mA/3.9 V, the contact resistance continued to decrease, ultimately stabilizing at an average value of 80 kΩ. This reveals that higher levels of resistive heating significantly improve the contact resistance performance of the thermal actuator. It is noteworthy that the doping surface of the 10 μm-thick Si layer is approximately 1–2 μm deep only as mentioned in the process handbook, and the actuator characterization revealed an out-of-plane displacement of ~0.5 μm [30]. However, operating with an overdrive value of 42 mA/3.9 V, following the main actuation voltage sufficient to close the switch, caused degradation in the Al heater. Similar characteristics were observed in the current study, where an overdrive power exceeding 205 mA/1.2 V resulted in damage to the Al heater with irreversible misalignment. This degradation is attributed to the formation of “hillocks” in the direction of current flow and the development of voids at the grain boundaries, as seen in Figure 11. Over time, this degradation led to reduced current flow until heater failure.

In addition to observing these alterations to the aluminum heater, other factors can impact the ability to maintain a consistent and stable contact resistance. As described in the next subsections, these factors include the sidewall contour of the MEMS switch, the contact force exerted at the interface, and the rise in the contact interface temperature resulting from localized heating by the Al heater. The sidewall contour can influence the mechanical alignment and contact quality, while the contact force affects the physical engagement between contact surfaces, leading to a reduced contact area. Additionally, the localized heating can lead to thermal expansion and variations in material properties, further complicating the stability of the contact resistance. The management techniques of these combined effects must be carefully elaborated to ensure the reliable and consistent performance of the MEMS switch, which is beyond the scope of this work.

### 5.1. Sidewall Topography Characterization

**Figure 12:**
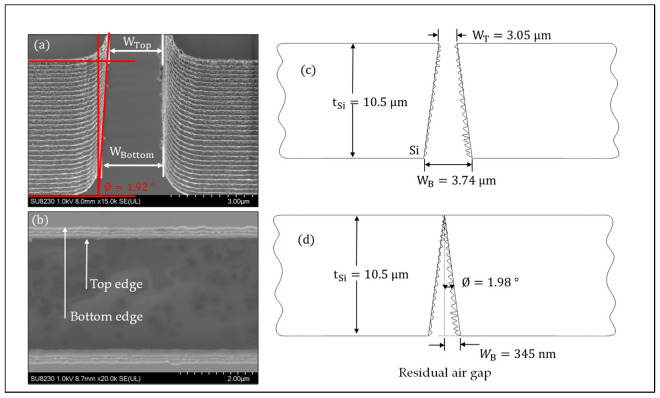
Switch contact interface: (**a**) cross-sectional view, (**b**) bottom view of the device, (**c**) sketched analysis of contact interface before activation, and (**d**) after activation.

The sidewall surface topography of the contacting surfaces was examined through SEM imaging analysis of the fabricated MEMS switches. These studies revealed that the scalloping outlines and asperity sizes of the sidewall surfaces varied between different fabrication runs, which could potentially limit the repeatability of the ohmic contact. Figure 12a presents an SEM image depicting the cross-sectional profile of the residual etching angle and the sidewall contours of the contact interface of the MEMS switches. The etch angles at the contact interface were measured to be approximately 1.92°. This etching angle resulted in a 608 nm air gap between the centers of the switching contacts upon initial contact. To further evaluate and validate the accuracy of the procedure used to extract the etching angle profile shown in Figure 12a, the MEMS die was mechanically flipped to observe the differences between the top edge-to-edge distance and the bottom edge-to-edge distance of the silicon structure. Figure 12b–d depicted a 690 nm residual etching after closure, which aligns well with the contact analysis. However, these factors could lead to inconsistencies in achieving well-aligned doped Si-to-Si contacts. The scalloping of the silicon sidewall is unavoidable due to the repeated alternation between DRIE etching and passivation cycles.

### 5.2. Manual Activation of the MEMS Switches with a Probe Needle

**Figure 13:**
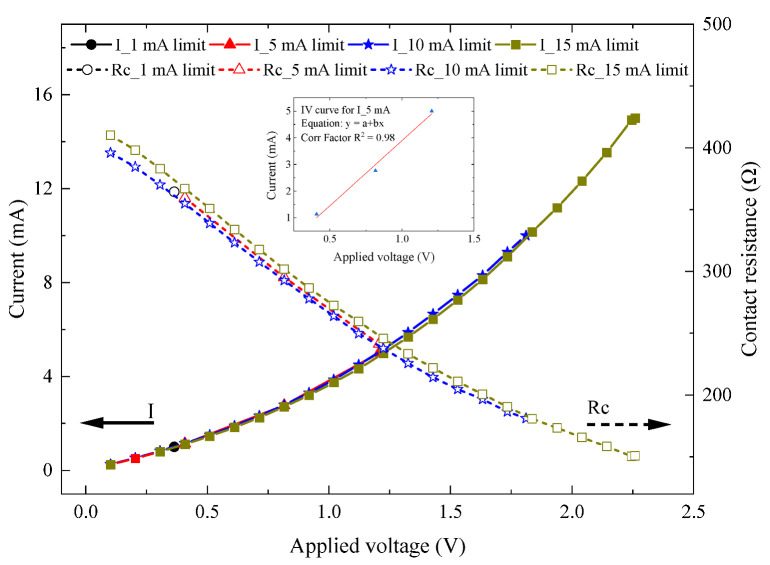
*I–V* plot of the switching contact and calculated contact on-resistance (R_ON_), with an inset picture showing the linear *I–V* curve for the 5 mA limit current.

The main objective of this experiment was to confirm the impact of high contact force and elevated temperature across the contact tip, resulting from Joule heating, on the value and the repeatability of the ohmic resistance of the MEMS switch. The on-state resistance was characterized using a Source Meter Unit (SMU) to obtain the *I–V* plot in Figure 13. The switches were manually triggered using a probe needle until a reasonable resistance was detected despite the less-controlled force applied with the needle. Once the switch closed, a voltage sweep was applied with a testing current (I) limited to 1, 5, 10, and 15 mA. The *I–V* plot in Figure 13 shows that the calculated resistance value (R_ON_) ranges from 150 Ω to 410 Ω, with a 5 min interval between each testing current. This method allowed for the evaluation of the switch’s performance by measuring the contact resistance at room temperature.

**Figure 14:**
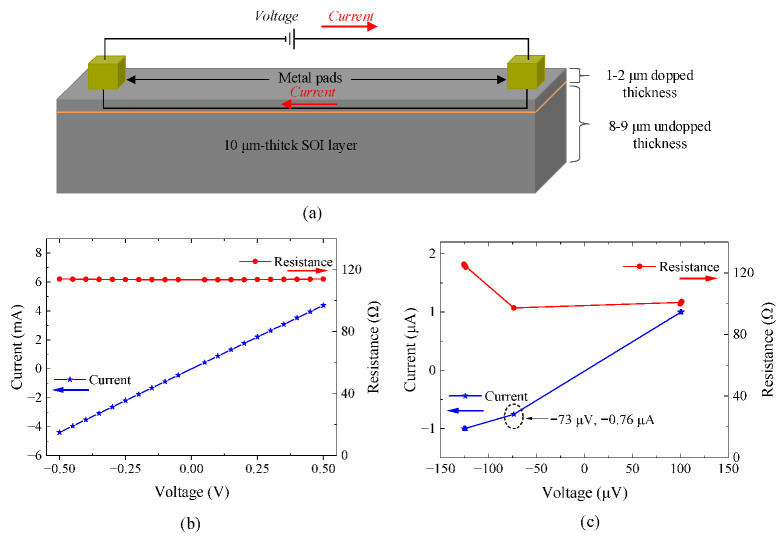
Silicon-Al contact: (**a**) schematic diagram of the bare silicon beam with an Al pad metal, (**b**) *I–V* plot in the range of −0.5 V to 0.5 V, and (**c**) *I–V* plot in the range of −150 μV to 150 μV.

As was observed in the previous graphs, despite the low Si-to-Si contact resistance obtained, for testing currents higher than 5 mA, the *I–V* relation was found to follow a less linear pattern. This could be due to the temperature generated by the higher testing currents. To better investigate this behavior, an additional experiment was conducted on a bare silicon beam with metal pads at its two ends, as shown in Figure 14a. The resistance of the beam was measured to be 116 Ω. This experiment is essential to understanding the behavior of the barrier between a uniformly surface-doped Si and a top metal (Al) pad, which are the major parts of our proposed Si-to-Si contact switch. The reliability and durability of the MEMS switches will significantly be influenced by the integrity of this junction, which depends on the type of metal used, the doping type and concentration of the Si, and the annealing process after metal deposition [35].

As illustrated by the *I–V* characteristic curve shown in Figure 14b, a voltage ranging from −0.5 to 0.5 V was applied on the Al pads to allow current through the Si beam and observe any possible Schottky barrier between the Si layer and the Al layer. As seen in Figure 14b, the current exhibited a linear relationship with the voltage, resulting in a constant resistance value. This linear response is expected, as surface doping of the Si layer for the PiezoMUMPs process (formerly SOIMUMPs) is very high (10^20^ cm^−3^) [36]. With such a significant surface doping concentration, carriers possess sufficient energy to efficiently tunnel through the inherent barrier between metals and semiconductors [37] and make the barrier behave like a ohmic one. However, applying a very low voltage in the range of −150 to 150 μV resulted in less ohmic contact where a nearby Schottky barrier was observed, as seen in Figure 14c. Nonetheless, with special annealing treatments and the careful selection of the metal, the height barrier responsible for the non-ohmic behavior could be reduced to near zero, and more ohmic behavior could be obtained [38].

Given the linear *I–V* curve observed in Figure 14b, the non-linear behavior seen in previous graphs is expected to stem largely from the silicon-to-silicon (Si-to-Si) contact when the switch is closed. This can be attributed to several factors. First, even though the silicon layer is heavily surface-doped, as shown in Figure 12 and Figure 14a, ensuring good and continuous contact between the 1–2 μm-deep doped regions of the two contacting edges is challenging. Etching processes can disturb the doping concentration near the contact edges. Moreover, the doping concentration is maximal at the top surface and gradually decreases toward the bottom surface of the Si layer. Therefore, as continuous force is applied to the switch to achieve good contact in the doped regions, the surface contact area of the less-doped regions also increases, potentially causing the non-linear response.

Second, residual etching materials used to pattern the silicon contact could form a thin sidewall coating, generating a barrier between the Si-to-Si contact when the switch is closed. Third, there is a possibility of the natural formation of a thin oxide layer on the edges of the Si contact, which could also act as a barrier [38]. The results and the foregoing analysis provide insights into necessary design optimizations for enhancing the contact behavior and, consequently, the longevity and repeatability of MEMS switches, particularly in applications requiring precise and stable electrical contact.

### 5.3. Comparison with State-of-the-Art MEMS Switches

To position this work within the context of state-of-the-art silicon-to-silicon (Si-to-Si) contact MEMS switches, Table 2 compares the proposed thermally actuated DC MEMS switch with previously reported devices, focusing specifically on Si-to-Si contact resistance. Among these, the switch presented in this work demonstrates a significant reduction in contact resistance, achieving values in the range of 150 Ω to 450 Ω, along with a satisfactory breakdown voltage. Additionally, despite the absence of heater optimization, which resulted in a shortened switch lifetime, the design maintains a reasonable electrical isolation between the heater and the signal line. Note that the high aspect ratio introduces a trade-off in the form of out-of-plane displacement.

## 6. Conclusions and Perspective

This study presented the design, fabrication, and characterization of an electro-thermally actuated Si-to-Si DC MEMS switch for power switching applications. The experimental results showed that upon closure, the switch exhibited an initial contact resistance of approximately 400 Ω and an average response time of 5.7 ms. The electrical contact resistance displayed non-linear behavior for currents ranging from 1 to 15 mA due to self-heating within the switch, while lower currents had negligible effects. These findings highlight the necessity of optimizing both mechanical force and thermal management to achieve consistent and reliable switch operation.

Detailed examination revealed that the etching angles of the sidewall contact rendered the normal contact force insufficient to adequately compress and polish the scallops and asperities of the sidewall, affecting contact resistance reliability and repeatability. Increasing the compressive load led to out-of-plane misalignment, affecting the contact resistance, due to doping confinement to the surface of the SOI device layer.

The impact of contact temperature on contact resistance was further investigated through a near-zero-temperature actuation test using manual contact via a micro-needle actuation. A linear *I–V* response was observed for currents below 5 mA, whereas higher currents induced non-linear responses due to Joule heating.

Importantly, the switching contacts sustained a maximum breakdown voltage of 350 V, owing to the actuator’s thermoelectric nature. The switch’s ability to withstand up to 350 V, coupled with low contact resistance, underscores its potential for demanding applications such as power systems and fault protection networks. Compared to other state-of-the-art MEMS switches, the proposed design offers good performance and a competitive breakdown voltage, despite challenges related to the heater lifetime.

Future work will focus on reducing the effects of Joule heating at the transmission line and optimizing the heater design to extend the switch’s lifespan.

## Figures and Tables

**Table 1:** Design dimensions of the thermal chevron actuator.

Geometry Parameter	Symbol	Value
Length of the chevron actuator	$L_{b}$	500 μm
Length of the shuttle	$L_{S}$	110 μm
Width of the chevron actuator	$W_{b}$	6 μm
Width of the chevron actuator	$W_{\mathrm{Si}}$	21 μm
Width of the Padoxide	$W_{\mathrm{OX}}$	11 μm
Width of Padmetal	$W_{\mathrm{Al}}$	5 μm
Angle of chevron beams	*β*	1.4°

**Table 2:** State-of-the-art MEMS switches compared to the proposed Si-to-Si MEMS switch.

Ref.	Actuation	Material	Motion	Actuation Voltage	$R_{ON}$	$V_{Breakdown}$
[26]	Electrostatic	Si-to-Si	Lateral		~35 kΩ	-
[29]	Electrostatic	Si-to-Si	In-plane	~18 V	No data	-
[39]	Electrostatic	Tungsten (W)	Out-of-plane	11.3 V	~7.5 kΩ	-
[40]	Electrostatic	Platinum (Pt)	In-plane	20 V	4.4-to-4.6 kΩ	-
This work	Electrothermal	Si-to-Si	In-plane	1.2 V	0.15 kΩ	350 V

## Data Availability

The original contributions presented in the study are included in the article; further inquiries can be directed to the corresponding author.

## References

[1] Pal J., Zhu Y., Lu J., Dao D., Khan F. (2016). High Power and Reliable SPST/SP3T RF MEMS Switches for Wireless Applications. IEEE Electron Device Lett..

[2] Keimel C., Claydon G., Li B., Park J.N., Valdes M.E. (2012). Microelectromechanical-Systems-Based Switches for Power Applications. IEEE Trans. Ind. Appl..

[3] Pothier A., Hitier S., El khatib M., Blondy P., Orlianges J.C., Champeaux C., Catherinot A., Vendier O., Cazaux J.L. MEMS DC contact micro relays on ceramic substrate for space communication switching network. Proceedings of the 2005 European Microwave Conference.

[4] Nguyen V.H., Aimaier N., Nobert G., Pham T., Constantin N., Blaquiere Y., Cowan G. (2022). A Reconfigurable Power System-in-Package Module using GaN HEMTs and IC Bare Dies on LTCC Substrate: Design—Implementation—Experiment and Future Directions. Proceedings of the 2022 20th IEEE Interregional NEWCAS Conference (NEWCAS).

[5] Maciel J., Majumder S., Lampen J., Guthy C. Rugged and reliable ohmic MEMS switches. Proceedings of the 2012 IEEE/MTT-S International Microwave Symposium Digest.

[6] Rebeiz G.M. (2003). RF MEMS.

[7] Song Y.-H., Han C.-H., Kim M.-W., Lee J.O., Yoon J.-B. (2012). An Electrostatically Actuated Stacked-Electrode MEMS Relay with a Levering and Torsional Spring for Power Applications. J. Microelectromech. Syst..

[8] Bakri-Kassem M., Mansour R.R. (2015). High Power Latching RF MEMS Switches. IEEE Trans. Microw. Theory Tech..

[9] Pal J., Zhu Y., Lu J., Dao D.V., Khan F. (2015). RF MEMS switches for smart antennas. Microsyst. Technol..

[10] Stefanini R., Chatras M., Blondy P., Rebeiz G.M. (2011). Miniature MEMS Switches for RF Applications. J. Microelectromech. Syst..

[11] Uvarov I., Kupriyanov A. (2019). Stiction-protected MEMS switch with low actuation voltage. Microsyst. Technol..

[12] Czaplewski D.A., Nordquist C.D., Dyck C.W., Patrizi G.A., Kraus G.M., Cowan W.D. (2012). Lifetime limitations of ohmic, contacting RF MEMS switches with Au, Pt and Ir contact materials due to accumulation of ‘friction polymer’ on the contacts. J. Micromech. Microeng..

[13] Newman H.S., Ebel J.L., Judy D., Maciel J. (2008). Lifetime Measurements on a High-Reliability RF-MEMS Contact Switch. IEEE Microw. Wirel. Compon. Lett..

[14] Ma B., You Z., Ruan Y., Chang S., Zhang G. (2016). Electrostatically actuated MEMS relay arrays for high-power applications. Microsyst. Technol..

[15] Zhang Y., Gong Z., Liu H., Liu Z. Power Handling Capability Enhanced RF MEMS Switch Using Modified-Width Cantilevers Structure. Proceedings of the 2021 IEEE 16th International Conference on Nano/Micro Engineered and Molecular Systems (NEMS).

[16] Iannacci J. (2017). RF-MEMS Technology for High-Performance Passives: The Challenge of 5G Mobile Applications.

[17] Sharma S., Nabavi S., Rabih A.A.S., Ménard M., Nabki F. (2023). Hybrid MEMS Actuator With 3 Degrees-of- Freedom for Efficient Planar Optical Switching. J. Microelectromech. Syst..

[18] Rabih A.A.S., Nabavi S., Ménard M., Nabki F. (2024). A 3 Degrees-of-Freedom Electrothermal Micro-Positioner for Optical Chip-to-Chip Alignment. J. Microelectromech. Syst..

[19] Rajagopalan J., Schmauder S., Chen C.-S., Chawla K.K., Chawla N., Chen W., Kagawa Y. (2019). Microelectromechanical Systems (MEMS)-Based Testing of Materials. Handbook of Mechanics of Materials.

[20] Agrawal V. A latching MEMS relay for DC and RF applications. Proceedings of the 50th IEEE Holm Conference on Electrical Contacts and the 22nd International Conference on Electrical Contacts Electrical Contacts, 2004.

[21] Shojaei-Asanjan D., Bakri-Kassem M., Mansour R.R. (2019). Analysis of Thermally Actuated RF-MEMS Switches for Power Limiter Applications. J. Microelectromech. Syst..

[22] Qiu J., Lang J., Schmidt M. (2003). An Electrothermally-Actuated Bistable MEMS Relay for Power Applications. Ph.D. Thesis.

[23] Daneshmand M., Fouladi S., Mansour R.R., Lisi M., Stajcer T. Thermally-actuated latching RF MEMS switch. Proceedings of the 2009 IEEE MTT-S International Microwave Symposium Digest.

[24] Mahameed R., Rebeiz G.M. (2010). A High-Power Temperature-Stable Electrostatic RF MEMS Capacitive Switch Based on a Thermal Buckle-Beam Design. J. Microelectromech. Syst..

[25] Wang L., Jin Y. (2017). A push-pull double-contact MEMS relay fabricated by MetalMUMPs process. Microsyst. Technol..

[26] Soon B.W., Qian Y., Ng E.J., Hong V.A., Yang Y., Ahn C.H., Kenny T.W., Lee C. (2015). Investigation of a Vacuum Encapsulated Si-to-Si Contact Microswitch Operated From −60 °C to 400 °C. J. Microelectromech. Syst..

[27] Zhu Y., Pal J. (2021). Low-Voltage and High-Reliability RF MEMS Switch with Combined Electrothermal and Electrostatic Actuation. Micromachines.

[28] Meszmer P., Hahn S., Hiller K., Wunderle B. (2019). Highly Miniaturized MEMS-Based Test Platforms for Thermo-Mechanical and Reliability Characterization of Nano-Functional Elements − Technology and Functional Test Results. Phys. Status Solidi (A).

[29] Bengashier M., Grech I., Casha O., Portelli B., Farrugia R. Mechanical Contact Type RF-MEMS Switches for Microwave Band Applications. Proceedings of the 2023 Symposium on Design, Test, Integration & Packaging of MEMS/MOEMS (DTIP).

[30] Shuaibu A.H., Nabki F., Blaquière Y. A MEMS Electrothermal Actuator Designed for a DC Switch Aimed at Power Switching Applications and High Voltage Resilience. Proceedings of the 2022 20th IEEE Interregional NEWCAS Conference (NEWCAS).

[31] Cowen A., Hames G., Glukh K., Hardy B. (2014). PiezoMUMPs™ Design Handbook.

[32] Coutu R.A., LaFleur R.S., Walton J.P.K., Starman L.A. (2016). Thermal Management Using MEMS Bimorph Cantilever Beams. Exp. Mech..

[33] https://aluminium-guide.com/melting-point-aluminium/.

[34] Science Corporation. https://science.xyz/docs/d/mems-piezo/index.

[35] Benouattas N., Tamaarat B., Bouabellou A., Halimi R., Mosser A. (1999). Electrical properties of Cr/Si(*p*) structures. Solid-State Electron..

[36] Ta Q. (2014). Local Synthesis and Direct Integration of Carbon Nanotubes into Microsystems for Sensor Applications. Ph.D. Thesis.

[37] https://cleanroom.byu.edu/ohmic-schottky.

[38] Card H.C. (1976). Aluminum—Silicon Schottky barriers and ohmic contacts in integrated circuits. IEEE Trans. Electron Devices.

[39] Osoba B., Almeida S.F., Sikder U., Ye Z.A., Hu X., Esatu T.K., Liu T.-J.K. (2020). Study of MEM Relay Contact Design and Body-Bias Effects on on-State Resistance Stability. J. Microelectromech. Syst..

[40] Grogg D., Drechsler U., Knoll A., Duerig U., Pu Y., Hagleitner C., Despont M. (2013). Curved in-plane electromechanical relay for low power logic applications. J. Micromech. Microeng..

